# Supplementary material for: VEGF in Tears as a Biomarker for Exudative Age-Related Macular Degeneration: Molecular Dynamics in a Mouse Model and Human Samples
Source: Int J Mol Sci. 2025 Apr 18;26(8):3855. doi: 10.3390/ijms26083855 (PMC12027898; doi:10.3390/ijms26083855)
Supplement: Supplementary file 1 [file ijms-26-03855-s001.zip › ijms-3563014-supplementary.pdf]

Supplementary Table 1: Antibodies and Primers Used in the Study

Antibodies Used in Immunohistochemistry (IHC) and Western Blot (WB)

Target Protein	Antibody Details	Dilution	Catalog No.	Manufacturer
CD206	Polyclonal Antibody	1:100	PA5-46994	Invitrogen
CD31	Mouse Monoclonal Antibody	1:50	558736	BD Biosciences Pharmingen
Actin	Rabbit Monoclonal Antibody	1:10,000	SAB5600204	Sigma-Aldrich Corp.
HIF-1α	Rabbit Polyclonal Antibody	1:500	NB100479	Novus Biologicals
VEGF	Mouse Monoclonal Antibody	1:200	SAB4200815	Sigma-Aldrich Corp.
NFκB p65	Mouse Monoclonal Antibody	1:200	sc-514451	Santa Cruz
Stat3	Mouse Monoclonal Antibody	1:200	sc-293151	Santa Cruz
p-Stat3	Mouse Monoclonal Antibody	1:200	sc-8059	Santa Cruz
p-RELA/NFκB p65	Mouse Monoclonal Antibody (Ser 536-specific)	1:200	sc-136548	Santa Cruz

qPCR Primers for SYBR® Green Gene Expression Analysis

Gene Symbol	Gene Name	Species	Unique Assay ID
Vim	Vimentin	Mouse	qMmuCED0046651
Rpl13a	Ribosomal Protein L13A	Mouse	qMmuCED0040629
Il6	Interleukin 6	Mouse	qMmuCED0045760
Vegfa	Vascular Endothelial Growth Factor A	Mouse	qMmuCED0040260
Ptgs2	Prostaglandin-Endoperoxide Synthase 2 (COX2)	Mouse	qMmuCED0047314
